# The role of maternal age & birth order on the development of unilateral and bilateral retinoblastoma: a multicentre study

**DOI:** 10.1038/s41433-022-01992-w

**Published:** 2022-03-31

**Authors:** Philippa Lloyd, Mark Westcott, Swathi Kaliki, Xunda Ji, Yihua Zou, Riffat Rashid, Sadia Sultana, Sadik Taju Sherief, Nathalie Cassoux, Rosdali Yesenia Diaz Coronado, Juan Luis Garcia Leon, Arturo Manuel Zapata López, Vladimir G. Polyakov, Tatiana L. Ushakova, Soma Rani Roy, Alia Ahmad, Lamis Al Harby, Jesse L. Berry, Jonathan Kim, Ashley Polski, Nicholas J. Astbury, Covadonga Bascaran, Sharon Blum, Richard Bowman, Matthew J. Burton, Allen Foster, Nir Gomel, Naama Keren-Froim, Shiran Madgar, Andrew W. Stacey, Ashik Mohamed, Marcia Zondervan, Mandeep S. Sagoo, Ido Didi Fabian, M. Ashwin Reddy

**Affiliations:** 1 Barts and The London School of Medicine and Dentistry, London, E1 2AD UK; 2 The Royal London Hospital, Barts Health NHS Trust, London, E1 1FR UK; 3 UCL Institute of Ophthalmology, London, EC1V 9EL UK; 4 Moorfields Eye Hospital NHS Foundation Trust, London, EC1V 2PD UK; 5 The Operation Eyesight Universal Institute for Eye Cancer, L V Prasad Eye Institute, Hyderabad, 500034 India; 6 Department of Ophthalmology, Xinhua Hospital, Shanghai Jiao Tong University School of Medicine, Shanghai, 200025 China; 7 Ocular Oncology, Ispahani Islamia Eye Institute and Hospital, Dhaka, 1215 Bangladesh; 8 Addis Ababa University, School of Medicine, Department of Ophthalmology, Addis Ababa, 3614 Ethiopia; 9 Institut Curie, Université de Paris Medicine Paris V Descartes, Paris, 75248 France; 10 Instituto Nacional de Enfermedades Neoplasicas, Lima, Peru; 11 Anglo American Clinic, Lima, 15073 Peru; 12 Head and Neck Tumors Department, SRI of Pediatric Oncology and Hematology of N.N. Blokhin National Medical Research Center of Oncology of Russian Federation, and Medical Academy of Postgraduate Education, Moscow, 115478 Russia; 13 Chittagong Eye Infirmary & Training Complex, Chittagong, 4202 Bangladesh; 14 The Children’s Hospital & the Institute of Child Health, Lahore, 54000 Pakistan; 15 Children’s Hospital Los Angeles, Keck School of Medicine, University of Southern California, Los Angeles, CA 90027 USA; 16 International Centre for Eye Health, London School of Hygiene & Tropical Medicine, London, WC1E 7HT UK; 17 Goldschleger Eye Institute, Sheba Medical Center, Tel Hashomer, Tel-Aviv University, Tel-Aviv, 52621 Israel; 18 Ophthalmology Department, Great Ormond Street Children’s Hospital, London, WC1N 3JH UK; 19 Division of Ophthalmology, Tel Aviv Sourasky Medical Center, Sackler Faculty of Medicine, Tel-Aviv University, Tel-Aviv, 39040 Israel; 20 Sackler Faculty of Medicine, Tel-Aviv University, Tel-Aviv, 39040 Israel; 21 Department of Ophthalmology, University of Washington, Seattle, WA 98195 USA; 22 Ophthalmic Biophysics (AMd), L V Prasad Eye Institute, Hyderabad, 500034 India; 23 NIHR Biomedical Research Centre for Ophthalmology at Moorfields Eye Hospital and UCL Institute of Ophthalmology and London Retinoblastoma Service, Royal London Hospital, London, EC1V 2PD UK; 24 Queen Mary University of London, London, E1 4NS UK

**Keywords:** Risk factors, Eye cancer

## Abstract

**Background/Objectives:**

Retinoblastoma is a common childhood intraocular malignancy, the bilateral form of which most commonly results from a *de novo* germline pathogenic variant in the *RB1* gene. Both advanced maternal age and decreasing birth order are known to increase the risk of *de novo* germline pathogenic variants, while the influence of national wealth is understudied. This cohort study aimed to retrospectively observe whether these factors influence the ratio of bilateral retinoblastoma cases compared to unilateral retinoblastoma, thereby inferring an influence on the development of *de novo* germline pathogenic variants in *RB1*.

**Subjects/Methods:**

Data from 688 patients from 11 centres in 10 countries were analysed using a series of statistical methods.

**Results:**

No associations were found between advanced maternal age, birth order or GDP per capita and the ratio of bilateral to unilateral retinoblastoma cases (*p* values = 0.534, 0.201, 0.067, respectively), indicating that these factors do not contribute to the development of a *de novo* pathogenic variant.

**Conclusions:**

Despite a lack of a definitive control group and genetic testing, this study demonstrates that advanced maternal age, birth order or GDP per capita do not influence the risk of developing a bilateral retinoblastoma.

## Introduction

Retinoblastoma is an intraocular malignancy, accounting for 3% of childhood cancers [1]. 95% of patients receive a diagnosis before the age of 5 and the majority of patients will receive a diagnosis before the age of 2 [2]. Globally, retinoblastoma is estimated to occur in 1 in 14,000–20,000 live births [3–11] with an annual incidence in children aged 0–4 years of roughly 11–13 per million [2, 12–14]. Roughly 60% of cases of retinoblastoma present unilaterally, while the remaining 40% present bilaterally [15, 16]. This bilateral phenotype generally has an earlier presentation than is seen in unilateral cases [17]. Retinoblastoma results from either a somatic or a germline pathogenic variant in the *RB1* tumour suppressor gene, causing inactivation [18]. Bilateral retinoblastoma is caused by a germline pathogenic variant, however 10–15% of unilateral cases also occur due to a germline pathogenic variant. Meanwhile, the remaining 85–90%, somatic changes occur to cause tumour development [19, 20]. It is estimated that 10% of all retinoblastoma cases are inherited while 30% of retinoblastoma cases occur due to a *de novo* germline pathogenic variant [21–23].

It has been determined that the number of new germline pathogenic variants occurring in a cell increases with parental age [24]. Both advanced maternal and paternal ages have been associated with various congenital syndromes, including Down syndrome and a number of cancers [25–28]. Genetic studies have suggested that a high proportion of *RB1* germline pathogenic variants are paternal in origin, while the influence of maternal age is debated [23, 29]. The effect of advanced maternal age on the risk of developing retinoblastoma is generally less studied than advanced paternal age.

The effect of birth order on the development of *de novo* germline pathogenic variants is relatively understudied. Birth order is thought to have some influence on the development of childhood cancers due to differences in in-utero hormone exposure between pregnancies, particularly between the first and the second [30, 31]. It is possible that a similar hormonal effect in seen in retinoblastoma, as one study found an increased risk of retinoblastoma in children who are first-born compared to those who are not [32]. Similarly, another study has shown decreasing risk of retinoblastoma with increasing birth order [33].

It is known that 80% of retinoblastoma cases worldwide are observed in lower- and middle-income countries [34]. An especially large proportion is seen in countries with high birth rates, possibly reflecting the increased odds of the occurrence of a *de novo* germline pathogenic variant with a higher number of conceptions [35].

This study tests the hypothesis that the ratio of unilateral versus bilateral retinoblastoma could be influenced by birth order, maternal age, or income status of the mother’s country. We chose GDP per capita of the country of birth of the mother as our measure. A multivariate logistic regression model is an ideal model to explore these effects as the dependent variable is retinoblastoma status, defined as either unilateral or bilateral.

## Materials and methods

This study was a collaboration of 11 retinoblastoma treatment centres located in 10 countries from five continents. The study was approved by the London School of Hygiene & Tropical Medicine Institutional Review Board (reference no. 15882), which granted a waiver of informed consent. Participating centres received ethics clearance in their respective countries according to local institutional guidelines. It was a one-year cross-sectional analysis that included all treatment-naïve retinoblastoma patients who presented to the participating centres from January 1, 2019 to December 31, 2019, and who were treated or offered treatment for retinoblastoma. All patients who had received prior treatment were excluded from this study.

Cases were classed as bilateral if they were bilateral at initial presentation, or if they originally presented unilaterally and subsequently developed a bilateral phenotype. Cases were only included if there was certainty of the status with regards to unilateral or bilateral disease at latest follow up, as this was the key dependent outcome variable in the analysis. The primary objective was to explore whether the ratio of unilateral versus bilateral retinoblastoma was influenced by birth order, maternal age or income status of the mother’s country.

Data were imported into SPSS version 26. Univariate analysis was performed to determine differences in the maternal characteristics between mothers with unilateral versus bilateral retinoblastoma, with respect to birth order, maternal age, and GDP per capita of mother’s residence. GDP per capita was chosen as it allowed us to assign each country a continuous variable which can be incorporated into a regression model. Where necessary, Log10 transformation was used to normalise data. This was performed for maternal age. Non-parametric equivalents were used for variables not normally distributed, such as birth order. A forward conditional logistic regression was built to investigate usefulness of the three predictive variables of birth order, maternal age, and GDP per capita in predicting retinoblastoma status as a binary outcome variable: “unilateral” or “bilateral”. Collinearity diagnostics was performed for maternal age (as Log10), BO, and GDP per capita in order to ensure no interdependence of these variables using a Variance Inflation Factor cut-off of <3.

## Results

Of the 689 total patients who presented to the participating centres, 461 possessed a unilateral retinoblastoma, while 228 (33.1%) possessed bilateral retinoblastoma (Table 1). In total, 8 subjects were missing maternal age data and 1 subject was missing birth order data. The full characteristics of the subjects can be seen in Table 1. All available data were analysed and inputted into the regression model.

**Table 1:** Laterality, maternal age and birth order characteristics of studied population.

Factor	Value	Bilateral *N* (%)	Unilateral *N* (%)	Total *N* (%)
Maternal age (years)	<20 years	19 (8.33)	36 (7.81)	55 (7.98)
	20–25	61 (26.75)	146 (31.67)	207 (30.04)
	26–30	77 (33.77)	154 (33.41)	231(33.53)
	31–35	46 (20.18)	76 (16.49)	122 (17.71)
	>35	22 (9.65)	44 (9.54)	66 (9.58)
	Missing maternal age data	3 (1.32)	5 (1.08)	8 (1.16)
	Total	228 (100.00)	461 (100.00)	689 (100.00)
Birth order	1	93 (40.79)	190 (41.21)	283 (41.07)
	2	79 (34.65)	185 (40.13)	264 (38.32)
	3	30 (13.16)	42 (9.11)	72 (10.45)
	4	14 (6.14)	25 (5.42)	39 (5.66)
	>4	12 (5.26)	18 (3.90)	30 (4.35)
	Missing birth order data	0 (0.00)	1 (0.22)	1 (0.15)
	Total	228 (100.00)	461 (100.00)	689 (100.00)

The univariate analysis showed that the distributions of birth order for bilateral versus unilateral retinoblastoma were very similar and did not differ significantly (Table 2). The median birth order for each group was 2. There was no significant difference in means of maternal age between the bilateral retinoblastoma group and the unilateral retinoblastoma group according to the one-way ANOVA (F = 0.336, *p* = 0.563). In addition, there was no significant difference in means of GDP per capita between the groups (F = 1.872, *p* = 0.172) (Table 2). In the forward stepwise conditional logistic regression, with a probability cut off for entry *p* < 0.05 for removal 0.1, none of the 3 inputted predictive factors entered the model, and only the constant entered the model (see Table 3). Collinearity diagnostics (SPSS) for maternal age (as Log10), birth order, and GDP per capita was performed. All three variables could reasonably be assumed to be independent of each other, using a Variance Inflation Factor cut-off of <3.

**Table 2:** Univariate analysis of predictive variables (means ± SD, or median & range).

Factor	Bilateral	Unilateral	Values
Mean maternal age^a^ ± SD	27.62 ± 5.84	27.37 ± 5.58	F = 0.336, *p* = 0.563
Median birth order and range	2 (1–9)	2 (1–8)	Independent Samples Mann–Whitney U Test *P* value = 0.376
Mean GDP per capita ± SD	7566.85 ± 11601.14	9275.02 ± 12861.30	F = 1.872, *p* = 0.172

^a^For ANOVA for maternal age, Log10 transform was used.

**Table 3:** Results of stepwise conditional logistic regression of collinearity of birth order, maternal age and GDP per capita and phenotypic bilateral retinoblastoma. Constant = −0.704.

Variable	Degrees of freedom	*P* value
Constant	1	0.001
Birth order	1	0.201
Log10 maternal age	1	0.534
GDP per capita	1	0.067

The ratio of unilateral versus bilateral disease, by country (GDP per capita) is presented in Table 4. The chi-square test showed no significant differences in the unilateral versus bilateral retinoblastoma between countries. For this analysis, UK and US figures were combined due to the small numbers in these groups and their similar GDP per capita.

**Table 4:** GDP per capita of each country in which there is a participating centre, the proportions of bilateral to unilateral cases in each country and the output of the Chi-square analysis.

Country	GDP per capita (US$)	Bilateral N (%)	Unilateral N (%)
Bangladesh	1698.3	51 (37.8)	84 (62.2)
China	9770.8	49 (29.5)	117 (70.5)
Ethiopia	772.3	25 (33.8)	49 (66.2)
France	41469.9	16 (32.7)	33 (67.3)
India	2010	45 (35.7)	81 (64.3)
Pakistan	1482.4	15 (50.0)	15 (50.0)
Peru	6941.2	8 (17.4)	38 (82.6)
Russia	11288.9	15 (35.7)	27 (64.3)
UK	42962.4	2 (14.3)	12 (85.7)
USA	62886.8	2 (28.6)	5 (71.4)
Total		228 (33.1)	461 (66.9)
Chi-Square Value	13.704		
Degrees of Freedom	8		
Significance	0.09		

## Discussion

This study found no association between any of the predictive factors we used. With regards to the effect of increasing maternal age as a risk factor for the development of bilateral retinoblastoma, the topic is controversial and the literature non-consistent. There are studies that support this hypothesis as well as those that refute it. Studies have investigated the contribution of both maternal age and paternal age to the development of a germline *de novo* pathogenic variant in *RB1*. Some studies have found an effect only of advanced paternal age but not of advanced maternal age, some have found an effect of both advanced paternal and maternal ages, some have found an effect of advanced maternal age only and some found an effect of neither [15, 16, 27, 36–42]. Full characteristics of these studies can be found in Supplemental Table 1. An advantage of this study is the large sample size. The relatively low incidence of retinoblastoma has meant that much of the pre-existing research uses small sample sizes, generally with fewer than 200 case subjects [15, 16, 27, 36, 40, 41]. Matsunaga et al. (1990) [38] used a similar sample size to this study, with 225 sporadic bilateral subjects and 408 sporadic unilateral subjects, and their findings of no effect of maternal age on the development of bilateral retinoblastoma support the findings of this study [38]. This may suggest that either chance or additional factors may be influencing results in studies reporting a difference. Conversely, it is also possible that this study, as well as Matsunaga et al. (1990) [38], did not detect an effect which is present. Further large studies would be required to investigate this.

A Swedish study by Yip et al. (2006) [37] had a similar sample size to this study and found an influence of maternal age over 40 years on the development of a retinoblastoma [37]. Our multivariate linear regression model assumes that any association for increased risk of bilateral retinoblastoma with increasing maternal age would be linear, which we did not find. However, our analysis cannot exclude a non-linear uptick in risk with advanced maternal age. A non-linear risk would be impossible to detect with our data, given the very small numbers of mothers above 40 in our study (4 subjects in the bilateral group and 9 subjects in the unilateral group). The data from Yip et al. (2006) [37] is also potentially flawed as laterality data was not used, therefore many cases would have had a unilateral phenotype, which is most likely due to a somatic pathogenic variant. Although the findings of this study cannot definitively conclude that there is no relationship between advanced maternal age and the development of a *de novo* germline pathogenic variant in *RB1*, they cast doubt over the findings of studies which have found an effect and call for much larger studies to be developed with appropriate inclusion criteria.

In addition, it cannot be excluded that observed effects of advanced maternal age may be a result of correlating behavioural confounders. For example, a study by Foix-L’Hélias et al. (2012) [43] observed a higher average maternal age in their retinoblastoma group than their controls, albeit with a smaller sample size than this study, but also observed that mothers over 35 years of age were significantly more likely to smoke than those under 35 years of age. Although this was adjusted for and the significant influence of advanced maternal age was still seen, it is very likely that additional behavioural differences between older and younger mothers exist which may not have been accounted for in some previous studies. This may provide an explanation for the large degree of controversy within the literature.

In our study, we found no effect of birth order on the ratio of unilateral versus bilateral retinoblastoma. However, our study does not have a control group, so the results must be interpreted with caution. A study by Laurvick et al. (2008) [32] suggests that the risk of developing retinoblastoma increases with decreasing birth order, while a study by Von Behren et al. (2011) [33] suggests that the risk of bilateral retinoblastoma is highest in first born children [32, 33]. It has been proposed that any influence of birth order on the risk of developing a *de novo* germline pathogenic variant is due to differences in in-utero hormone exposure. For example, it has been found that circulating free oestradiol is significantly higher in a first pregnancy than at a comparable time point in the second, which is known to increase the risk of testicular cancer in adulthood [31, 44]. It is possible that the effect of birth order is very small, and only increases marginally with first born children. As we did not have a control group, and only relied on the ratio bilateral to unilateral retinoblastoma, then perhaps our sample size was underpowered to detect this effect and our finding is a type II statistical error. It is noteworthy that Laurvick et al. (2008) [32] did detect a birth order effect, but only had 38 retinoblastoma patients, with a very large control group of 576,352 controls. Laurvick et al. (2008) [32] did not distinguish laterality or use genetic testing so their observed effect may be due to differences in factors affecting inheritance of an *RB1* pathogenic variant. In addition, the reported hazard ratio had a 95% confidence interval between 0.46–1.64. As this includes 1.0, the results should be interpreted with caution. However, both Laurvick et al. (2008) [32] and Von Behren et al. (2011) [33] used national birth data as a control, while this study had no disease-free control group. Full characteristics of these studies can be found in Supplemental Table 2.

Our study methodology has some advantages. Firstly, unlike Laurvick et al. (2008) [32], we investigated birth order on the risk of developing a bilateral retinoblastoma, as against unilateral retinoblastoma, in order to more specifically investigate risk factors for *de novo* germline pathogenic variants. Another strength of our study is that multivariate logistic regression allowed us to adjust for the effects of several possible predictor variables on the risk of bilateral retinoblastoma, both with negative correlation (e.g. birth order) or positive correlation (maternal age). Our model assumptions were valid as we tested for collinearity of predictor variables and demonstrated that they were independent and not correlated.

We failed to find significant inter-country differences, according to GDP per capita, between the ratio of unilateral versus bilateral retinoblastoma tested both with non-parametric (Chi-square) and parametric statistics (logistic regression model). There has previously been little research into whether factors associated with national income affect the development of *de novo* germline pathogenic variants. It is known that differences in healthcare delay diagnosis in lower-income countries and result in poorer prognosis and a higher incidence of death before diagnosis [34]. This limitation is supported as the higher mortality before child-bearing age decreases the incidence of familial retinoblastoma in these countries [34]. This lack of detection of retinoblastoma patients in many low-income countries present a challenge for any future studies in detecting differences in rates of *de novo* germline pathogenic variants in *RB1*.

Limitations of this study are that the data is retrospective, and that we do not have a control group of disease-free subjects. Although this would have been ideal, the method of using unilateral retinoblastoma patients as a comparator against bilateral retinoblastoma patients has been used by multiple previous studies [15, 39, 40]. However, there may still be differences in the investigated factors between unilateral retinoblastoma patients and the general population so they cannot be fully equated. Additionally, a lack of genetic testing allowing the distinction between subjects with *de novo* germline pathogenic variants, de novo somatic pathogenic variants and familial pathogenic variants means that the groups are not fully distinct due to a degree of overlap. As all germline *de novo* cases present bilaterally, any factor which encourages the development of a *de novo* germline pathogenic variant in *RB1* should create a higher level of bilateral retinoblastoma within a population. 10–15% cases in the unilateral group will be germline in origin and would ideally be included in the case group, however due to a lack of genetic testing these cases cannot be distinguished from those with somatic origin [19]. This may produce a type II error, rather than induce a false positive result. Although any factor causing an increased risk of developing a *de novo* germline pathogenic variant in *RB1* should cause an increase in the number of bilateral retinoblastoma patients, one cannot be certain that the increase is not due to a change in the small proportion of the patients with somatic pathogenic variants in *RB1* within the group. Therefore, a large study with genetic testing within the methodology should be used to draw final conclusions.

In addition to the large sample size, an advantage of this study is that the statistics are robust. Also, the data from all of the participating centres is near-complete and therefore may be viewed as an accurate representation of the retinoblastoma patients from their respective areas. Another limitation is that there was family history of retinoblastoma in three children (one from a high-income country and two from lower-middle income countries) and they were detected to have retinoblastoma after routine fundus screening. We attempted to exclude all other familial cases, however these cases may hold some influence over the results.

## Conclusions

To conclude, this large study found no association between any of the factors tested, which were increasing maternal age, birth order or GDP per capita on the risk of developing de novo germline bilateral retinoblastoma compared to unilateral retinoblastoma. This therefore suggests that these factors do not contribute to the development of a *de novo* germline pathogenic variant in *RB1*. However, this study did not use genetic testing so could only use laterality data as an indication of origin of the pathogenic variant i.e. inherited, somatic or *de novo*. Due to the degree of conflicting results in the literature, larger studies using genetic testing of patients, and healthy controls should be used in order to reach an unequivocal conclusion.

## Summary

### What was known before

- Both advanced maternal age and decreasing birth order are known to increase the risk of *de novo* germline pathogenic variants.
- The influence of national wealth is understudied.
- Some studies report an influence of maternal age and birth order on the risk of developing a *de novo* germline pathogenic variant in *RB1*, causing bilateral retinoblastoma, while others report no influence.
- There is a great deal of controversy.

### What this study adds

- This study reports no statistically significant influence of these factors on the risk of developing a *de novo* germline pathogenic variant in *RB1*.
- This study uses one of the largest and most diverse samples to date.

## Supplementary information

Supplemental Table 1

Supplemental Table 2
