# Supplementary material for: The role of maternal age & birth order on the development of unilateral and bilateral retinoblastoma: a multicentre study
Source: Eye (Lond). 2022 Mar 31;37(5):966–70. doi: 10.1038/s41433-022-01992-w (PMC10050160; doi:10.1038/s41433-022-01992-w)
Supplement: Supplementary file 1 — Supplemental Table 1 [file 41433_2022_1992_MOESM1_ESM.docx]

| **Authors** | **Study Sample** | **Control Sample** | **Findings** |
| --- | --- | --- | --- |
| **Pellié et al., 1973 ^39^** | 155 patients with sporadic bilateral RB at Hopital des Enfants Malades (France) | 289 patients with sporadic unilateral RB taken from French Vital Statistics | Increased paternal age associated with sporadic bilateral retinoblastoma |
| **Bunin et al., 1989 ^42^** | 201 sporadic bilateral RB patients and sporadic unilateral RB patients with confirmed constitutional 13q deletion from Children’s Cancer Study Group member hospitals (U.S.A) | 201 healthy individuals selected by random digit dialling and pair-matching | No significant effect but a trend of advanced maternal and paternal ages in retinoblastoma patients |
| **Der Kinderen et al., 1990 ^40^** | 104 patients with sporadic retinoblastoma (bilateral or unilateral with no family history). Data taken from Netherlands General Association for the Prevention of Blindness Registry | 257 patients with sporadic non-hereditary retinoblastoma (no family history, and unilateral disease), taken from Netherlands General Association for the Prevention of Blindness Registry, and general population data of the Netherlands | Significantly higher maternal and paternal ages in patients with sporadic hereditary retinoblastoma than in the general population |
| **Matsunaga et al., 1990 ^38^** | 225 patients with sporadic bilateral retinoblastoma and 10 sporadic cases of chromosome deletion or translocation of 13q14 gene, taken from a nationwide retinoblastoma registry in Japan | Japanese general population data | No evidence of maternal or paternal age effects |
| **Moll et al., 1996 ^27^** | 178 sporadic hereditary retinoblastoma patients (classified as when a bilateral retinoblastoma has occurred, a family history for retinoblastoma is known or determined by chromosomal/DNA analysis) taken from Dutch Retinoblastoma Registry | 437 sporadic non-hereditary retinoblastoma patients (those not meeting criteria for sporadic hereditary classification) taken from Dutch Retinoblastoma Registry and general population data of the Netherlands | Higher mean maternal and paternal ages in sporadic hereditary retinoblastoma than general population |
| **Yip et al., 2006 ^37^** | 226 retinoblastoma patients (diagnosed by International Classification of Disease (ICD-7) or pathological anatomic diagnosis) taken from Swedish Cancer Registry | General population data of Sweden | Maternal age of 40 and over associated with increased risk of retinoblastoma |
| **Johnson et al., 2009 ^36^** | 660 retinoblastoma patients taken from population-based state cancer registries (U.S.A.). Unilateral and bilateral cases examined separately. | Randomly selected individuals from each state’s birth registry in ratios to cases that varied from 1:1 to 10:1, matched via frequency matching (individual matching used in one state) | No evidence of maternal or paternal age effects |
| **Mills et al., 2011 ^15^** | 46 patients with ‘de *novo* germline pathogenic variant’ defined as those with bilateral retinoblastoma and no or unknown family history, taken from National Cancer Institute Cohort of Retinoblastoma Survivors (U.S.A.) | General population data of U.S.A. | Weak effect of paternal age on risk of de *novo* retinoblastoma |
| **Saremi et al., 2014 ^16^** | 120 randomly selected retinoblastoma cases presenting at Mahak Hospital (Iran) | 120 healthy individuals from Mofid Hospital (Iran) | Advanced maternal age associated with increased risk of retinoblastoma |
| **Heck et al., 2015 ^41^** | 280 sporadic retinoblastoma cases (185 unilateral and 95 bilateral) from Wills Eye Hospital in Philadelphia, or by the Children’s Oncology Group (COG) (U.S.A) | 155 case-matched controls, nominated by the family | Some indication of association of advanced maternal age and risk of retinoblastoma |
| **Lloyd et al. 2022** | 228 bilateral retinoblastoma patients attending to 11 centres in 10 countries over one year | 461 unilateral retinoblastoma patients attending to 11 centres in 10 countries over one year | No association of advanced maternal age with risk of developing bilateral retinoblastoma |
